# Health state utility values in people living with HTLV-1 and in patients with HAM/TSP: The impact of a neglected disease on the quality of life

**DOI:** 10.1371/journal.pntd.0008761

**Published:** 2020-10-16

**Authors:** Carolina Rosadas, Tatiane Assone, Marina Yamashita, Adine Adonis, Marzia Puccioni-Sohler, Marisa Santos, Arthur Paiva, Jorge Casseb, Augusto C. P. Oliveira, Graham P. Taylor

**Affiliations:** 1 Section of Virology, Department of Infectious Disease, Imperial College London, London, United Kingdom; 2 Faculdade de Medicina / Instituto de Medicina Tropical de São Paulo, Universidade de São Paulo, São Paulo, Brazil; 3 Universidade Federal do Estado do Rio de Janeiro, Rio de Janeiro, Brazil; 4 National Centre for Human Retrovirology, St. Mary’s Hospital, Imperial College Healthcare NHS Trust, London, United Kingdom; 5 Universidade Federal do Rio de Janeiro, Rio de Janeiro, Brazil; 6 Instituto Nacional de Cardiologia, Núcleo de Avaliação em Tecnologia em Saúde, Rio de Janeiro, Brazil; 7 Universidade Federal de Alagoas, Hospital Universitário Prof. Alberto Antunes, Brazil; 8 Instituto de Infectologia Emílio Ribas, Universidade de São Paulo, São Paulo, Brazil; Northeastern University, UNITED STATES

## Abstract

Background: HTLV-1 is a neglected sexually transmitted infection despite being the cause of disabling neurological disease HTLV-1-associated myelopathy/tropical spastic paraparesis (HAM/TSP). There is no treatment for this infection and public health policies are essential to reduce its transmission. However, there are no data to support adequate cost-effective analysis in this field. The aim of this study was to obtain health state utility values for individuals with HAM/TSP and HTLV-1 asymptomatic carriers (AC). The impact of both states on quality of life (QoL) is described and compared to other diseases. Methods: A cross-sectional observational study of 141 individuals infected with HTLV-1 (79 with HAM/TSP and 62 AC) from three Brazilian states (Rio de Janeiro, São Paulo and Alagoas) and from the United Kingdom. Participants completed a validated general health questionnaire (EQ-5D, Euroqol) from which country specific health state utility values are generated. Clinical and epidemiological data were collated. Principal findings: Health state utility value for HAM/TSP was 0.2991. QoL for 130 reported clinical conditions ranges from 0.35 to 0.847. 12% reported their quality of life as worse as death. Low QoL was associated with severity rather than duration of disease with a moderate inverse correlation between QoL and Osame’s Motor Disability Score (-0.4933) Patients who are wheelchair dependent had lowest QoL whilst those still walking unaided had the highest. AC also reported impaired QoL (0.7121) compared to general population. Conclusion: HTLV-1 and its associated neurological disease has a marked impact on QoL. This study provides robust data to support the development of cost-utility analysis of interventions for HTLV-1.

## Author summary

HTLV-1 is a life-long persistent infection with no curative treatment available. It can cause a disabling neurological disease (HAM/TSP). Public policies targetting HTLV-1 prevention are important. Cost-utility studies are essential to identify which policies should be implemented. These studies have been hampered by the lack of information about health state utility values in patients with HAM/TSP and asymptomatic carriers (AC). These values represent the quality of life (QoL) and may vary from 0 (death) to 1 (best health state). We determined these values for AC (0.7121) and HAM/TSP patients (0.2991) using a self-administered questionnaire in patients from Brazil and UK. QoL in patients with HAM/TSP is lower than that reported for more than 130 other conditions with such data, including multiple sclerosis, neuropathic pain, Parkinson’s disease and HIV infection. For 12% of patients with HAM/TSP QoL was worse than death. Disease severity, rather than duration of disease, is associated with decreased QoL in HAM/TSP. HTLV-1 AC have impaired QoL compared to the general population in both countries. The data presented here will inform proper economic analysis in order to identify cost-effective policies. This is especially important for low- and middle-income countries where HTLV-1 prevalence is high and resources are limited.

## Introduction

Human T cell lymphotropic virus type 1 (HTLV-1) is a retrovirus that is transmitted during sexual intercourse, by contact with infected blood and tissues and from mother-to-child, mainly through breastfeeding. A conservative estimate concluded that at least 5–10 million individuals are currently living with HTLV-1 throughout the world and it is clear that some countries have a high number of infections based on population size and high HTLV-1 seroprevalence [1]. HTLV-1 infection is commonly associated with disadvantaged populations, be they in low- and middle-income countries or marginalised groups such as immigrants, sex workers and injecting drug users within otherwise prosperous nations. Indeed, the prevalence can reach 12% of the rural population in Gabon[2], 45% of the indigenous population of central Australia[3], and up to 4% of pregnant women from some Latin-American countries, such as Jamaica and French Guyana[4–6]. HTLV-1 is associated with a broad range of clinical disorders, from the aggressive leukaemia (adult T cell leukaemia/lymphoma, ATL), the disabling neurological disease (HTLV-1-associated myelopathy / tropical spastic paraparesis (HAM/TSP)), and other inflammatory conditions, such as uveitis, infective dermatitis and polyarthritis through to increased morbidity and mortality with co-infections. Recently, a meta-analysis revealed that HTLV-1 infection is associated with a 1.6-fold higher risk of early death due to any cause[7]. There is no curative treatment for this lifelong persistent infection. The impact of HAM/TSP on quality of life (QoL) is evident [8–17] and the need for preventive measures to avoid HTLV-1 transmission is clear. Despite this HTLV-1 infection remains neglected [18,19]. Screening of blood donors, although effective in preventing iatrogenic infection, is not implemented globally. National antenatal screening testing has been implemented only in Japan despite the high rate of infection observed in pregnant women in many countries. In order to implement public policies for HTLV-1, economic evaluation is essential to assess the cost-effectiveness of each policy in each setting.

Cost-effectiveness evaluations are based on the comparison of both costs and effects of the technology/policy that are under appraisal and the relevant comparison, for example, with and without an intervention (such as screening of blood donors for HTLV-1/2). The effects of an intervention can be measured by different variables, such as the number of infections prevented, lives saved or years of life lost averted [20]. The Euroqol five dimension questionnaire (EQ-5D) is the preferred method for cost-utility analysis for several health technology assessment organizations, including the National Institute for Health Care Excellence (NICE)[21]. EQ-5D is a generic questionnaire that generates a unique general health state. Then, the index score (EQ-5D Index or health state utility value (HSUV)) can be calculated applying the country-specific utility scoring algorithm [22]. The algorithm also reflects a society’s preference for each health state. EQ-5D Index can be used to generate quality-adjusted life years (QALYs), considered extremely important in cost-utility analysis [20,23,24]. Currently, there is a list of EQ-5D Indices for several diseases in different countries [25,26], however the EQ-5D Index has not been applied to HAM/TSP. Previous cost-effectiveness studies in the HTLV-1 field used values obtained from patients with chronic diseases (not HAM/TSP) [27] or specialist opinion [28] neither of which are truly representative. Recently, cost-utility study to evaluate screening of blood donors for HTLV-1 in South Africa was hampered by the lack of utilities data for HTLV-1[29].

Therefore, in order to produce more robust data for further economic analysis and to better understand the impact of HTLV-1 infection and its associated neurological disease on patients’ quality of life, health state utility values were determined for HTLV-1 infected individuals with either asymptomatic infection or HAM/TSP from both United Kingdom (UK) and Brazil. The impact of clinical and epidemiological data on the obtained values were analysed. The results were compared with published values for different diseases in both countries.

## Methods

All individuals included in this observational cross-sectional study had HTLV-1 infection confirmed by Western Blot and/or PCR and had negative serology for HIV. The participants consisted of consecutive patients attending different clinical settings from UK (National Centre for Human Retrovirology, St. Mary’s Hospital, London) and Brazil (Hospital Universitario Gaffreé Guinle (HUGG), Rio de Janeiro (RJ), Instituto de Infectologia Emílio Ribas, São Paulo (SP), Hospital Universitário Professor Alberto Antunes, Universidade Federal de Alagoas (AL)). All patients with HAM/TSP were undergoing symptomatic treatment. Some had received immunosuppressive therapy (methylprednisolone pulse therapy every 45 days, oral daily prednisolone or oral weekly methotrexate).

The Brazilian and English version of Euroqol 5D questionnaire (EQ-5D) were provided by the Euroqol group. The Brazilian version consisted of EQ-5D-3L while the EQ-5D-5L version was used in the UK. Although the EQ-5D-5L is the most up to date version and was developed to increase the questionnaire’s sensitivity, this version has not been validated in Brazil. EQ-5D has five dimensions: mobility, self-care, usual activities, pain / discomfort and anxiety/depression. In the EQ-5D-3L version for each dimension the patient has three options regarding their actual health state: (1) no problem, (2) some or moderate problem and (3) extreme problem, while for the EQ-5D-5L version patients have five options: (1) no problem, (2) slight problems, (3) moderate problems, (4) severe problems and (5) unable or extreme problems. The combination of the three or five possible answers in the five domains results in a health state (for example: 32112). Then, the observed health state obtained for each patient is used to calculate the EQ-5D Index, according to values obtained for the Brazilian [30] or UK population [31]. The Index which is generated is weighted for the value attributed to each domain by their country’s population. The Indices generated by EQ-5D-3L and EQ-5D-5L are comparable. EQ-5D Index varies from 0 (death) to 1 (best state of health). Values lower than zero can be obtained and represent a quality of life which is worse than death[22].

A visual analogue scale (VAS) is also part of the EQ-5D questionnaire and consists of a self-rating scale which represents the patient`s overall health. It ranges from 0 (worst imaginable health state) to 100 (best imaginable health state), and each individual should mark on the scale the value that best represents their current health state.

Clinical and demographic information, consisting of gender, age, educational level, duration of symptoms, necessity of walking aid and Osame Motor Disability Score (OMDS)[32] were also analysed. Osame Motor Disability Score ranges from 0 up to 13, where zero represents a fully ambulant patient and 13 reflects patients that are not able to move even their toes [32].

A descriptive statistical analysis for each dimension is shown. The percentage of individuals reporting problems was compared to previously published data for general population from Brazil and UK, that was also obtained using EQ-5D questionnaire[30,33]. The EQ-5D Index scores from patients with HAM/TSP were compared to HTLV-1 asymptomatic carriers (AC) and the UK patients were compared with the Brazilian. D`Agostino and Pearson omnibus normality test was used to verify whether data had a Gaussian distribution. Unpaired t test and Mann Whitney test were used to compare groups, while Pearson or Spearman test were used to test if there were significant correlations between data for those with gaussian and non-gaussian distribution, respectively. The results were considered statistically significant if p < 0.05.

### Ethics statement

The clinical data from the UK patients, including EQ-5D, were gathered as part of routine clinical care and their use in this study was in accordance with NRES guidelines. In Brazil the data were collated from two separate studies approved by the respective local ethical committees: (Emílio Ribas Ethical Committee and the HUGG/ Universidade Federal do Estado do Rio de Janeiro Research Ethics Committee Board under No. 40/07). All data included in this study was anonymized.

## Results

### Clinical and Epidemiological data

A total of 141 individuals infected with HTLV-1 from three Brazilian states and from the UK were included in this study (after excluding five HIV-co-infected patients, two individuals that did not complete the whole EQ-5D questionnaire and one patient with HAM and concomitant ATL). The studied population comprises 79 patients with HAM/TSP: 20 from the neuroinfection outpatient clinic at Hospital Universitario Gaffreé Guinle (HUGG), Rio de Janeiro (RJ), 17 attending the Instituto de Infectologia Emílio Ribas, São Paulo (SP), 20 attending Hospital Universitário Professor Alberto Antunes, Universidade Federal de Alagoas (AL) and 22 from the National Centre for Human Retrovirology, St. Mary’s Hospital, London (UK) and 62 asymptomatic individuals living with HTLV-1 (36 from AL, 8 from SP and 18 from UK). A summary of the clinical and demographic characteristics of the studied individuals is presented in Table 1. There was no significant difference regarding gender between the studied groups (HAM/TSP and AC). The median age of HAM patients was significantly higher than AC individuals (except for UK patients only).

**Table 1:** Clinical and demographic characteristics of the studied population.

	Brazil	UK	Total
**Age** (Median, Min-Max)			
**HAM/TSP**	57 (17–77)*	61 (33–79)	59 (17–79)*
**AC**	47 (18–83)	55.5 (31–77)	50 (18–83)
**Gender**			
**HAM/TSP**			
Male n (%)	14 (24.6)	10 (45.5)	24 (30.4)
Female n (%)	43 (75.4)	12 (54.5)	55 (69.6)
**AC**			
Male n (%)	18 (40.9)	3 (16.7)	21 (33.9)
Female n (%)	26 (59.1)	15 (83.3)	41 (66.1)
**HAM/TSP**			
**Disease duration** years Mean (SD)	14.4 (9.1)	14.4 (8.3)	14.4 (8.8)
**Immunosuppressive treatment**			
Yes n (%)	28 (49.1)	13 (59.1)	41(51.9)
No n (%)	29 (50.9)	9 (40.9)	38 (48.1)
**Walking aid**			
Unaided n (%)	6 (10.5)	5 (23.8)	11 (14.1)
Unilateral n (%)	9 (15.8)	5 (23.8)	14 (17.9)
Bilateral n (%)	24 (42.1)	7 (33.4)	31 (39.7)
Wheelchair n (%)	18 (31.6)	4 (19)	22 (28.2)
**OMDS** (Median, Min-Max)	7 (2–10)	7 (1–11)	7 (2–10)

UK: United Kingdom, HAM/TSP: HTLV-1 associated myelopathy; AC: HTLV-1 asymptomatic carrier; OMDS: Osame Motor Disability Score *p<0.05

### QoL in patients with HAM/TSP and in HTLV-1 asymptomatic carriers

**Fig 1:**
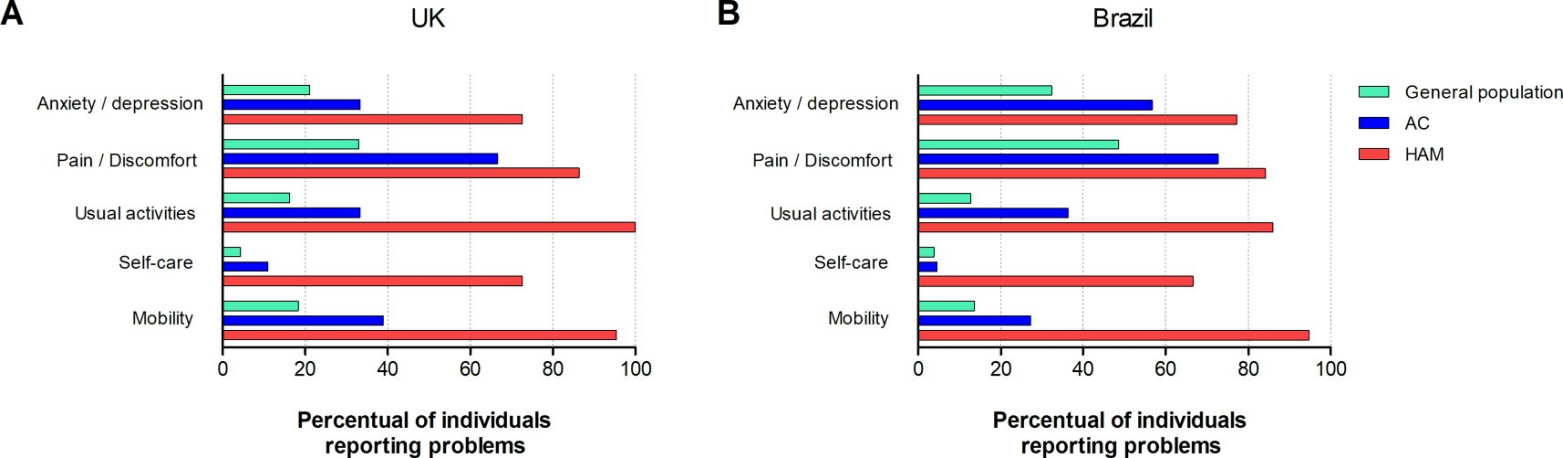
Percentage of individuals reporting some problems in each domain according to clinical status and compared with general population. A) UK (general population data according to Sullivan et al 2011 and Szende et al 2014); B) Brazil (general population data according to Santos et al 2016).

The great majority of patients with HAM/TSP reported reduced QoL with half (50·6%) reporting some degree of disturbance in each of the five domains. In Brazil, mobility was the main domain affecting the quality of life of patients with HAM/TSP with 95% reporting a problem, followed by impact on usual activities (86%), pain / discomfort (84%), anxiety/ depression (77%) and self-care (67%). The most frequently affected domain among UK patients with HAM/TSP was usual activities (100% identified some degree of problem), followed by mobility (95%), pain/discomfort (86%) and anxiety/depression (73%) and self-care (73%). Amongst HTLV-1 AC pain / discomfort is the most frequently affected domain in both countries (Fig 1 and Table 2). Rates of anxiety/depression in ACs differed by country with 78% reporting no or only slight problem in the UK compared with 57% of AC in Brazil reporting moderate or severe anxiety/depression. Compared with published data from the general populations of Brazil and UK, it is evident that HAM/TSP is associated with impaired quality of life in all five domains. Moreover, compared with the general population a higher percentage of reportedly asymptomatic HTLV-1 carriers had some problem in all domains (Fig 1). Self-care was the only domain in which AC and the general population had similar results.

**Table 2:** Frequency of HTLV-1 infected individuals reporting problems and its severity in each of five domains of Euroquol questionnaire, according to clinical status (AC or HAM/TSP) and country of origin.

	Mobility	Self-care	Usual activities	Pain / Discomfort	Anxiety / Depression % (n)
	% (n)	% (n)	% (n)	% (n)	
**AC**					
**Brazil**					
No problem	72.7 (32)	95.5 (42)	63.6 (28)	27.3 (12)	43.2 (19)
Some or moderate problem	25 (11)	4.5 (2)	34.1 (15)	59.1 (26)	40.9 (18)
Extreme problem	2.3 (1)	0 (0)	2.3 (1)	13.6 (6)	15.9 (7)
**UK**					
No problem	61.1 (11)	88.9 (16)	66.7 (12)	33.3 (6)	66.7 (12)
Slight problems	33.3 (6)	11.1 (2)	22.2 (4)	38.9 (7)	11.1 (2)
Moderate problems	0 (0)	0 (0)	11.1 (2)	22.2 (4)	22.2 (4)
Severe problems	5.6 (1)	0 (0)	0 (0)	0 (0)	0 (0)
Unable/ extreme problems	0 (0)	0 (0)	0 (0)	5.6 (1)	0 (0)
**HAM/TSP**					
**Brazil**					
No problem	5.3 (3)	33.3 (19)	14 (8)	15.8 (9)	22.8 (13)
Some or moderate problem	63.2 (36)	57.9 (33)	61.4 (35)	50.9 (29)	52.6 (30)
Extreme problem	31.6 (18)	8.8 (5)	24.6 (14)	33.3 (19)	24.6 (14)
**UK**					
No problem	4.6 (1)	27.3 (6)	0 (0)	13.6 (3)	27.3 (6)
Slight problems	9.1 (2)	31.8 (7)	31.8 (7)	18.2 (4)	45.4 (10)
Moderate problems	13.6 (3)	22.7 (5)	18.2 (4)	22.7 (5)	13.6 (3)
Severe problems	31.8 (7)	13.6 (3)	36.4 (8)	31.8 (7)	9.1 (2)
Unable / extreme problems	40.9 (9)	4.6 (1)	13.6 (3)	13.6 (3)	4.6 (1)

**Fig 2:**
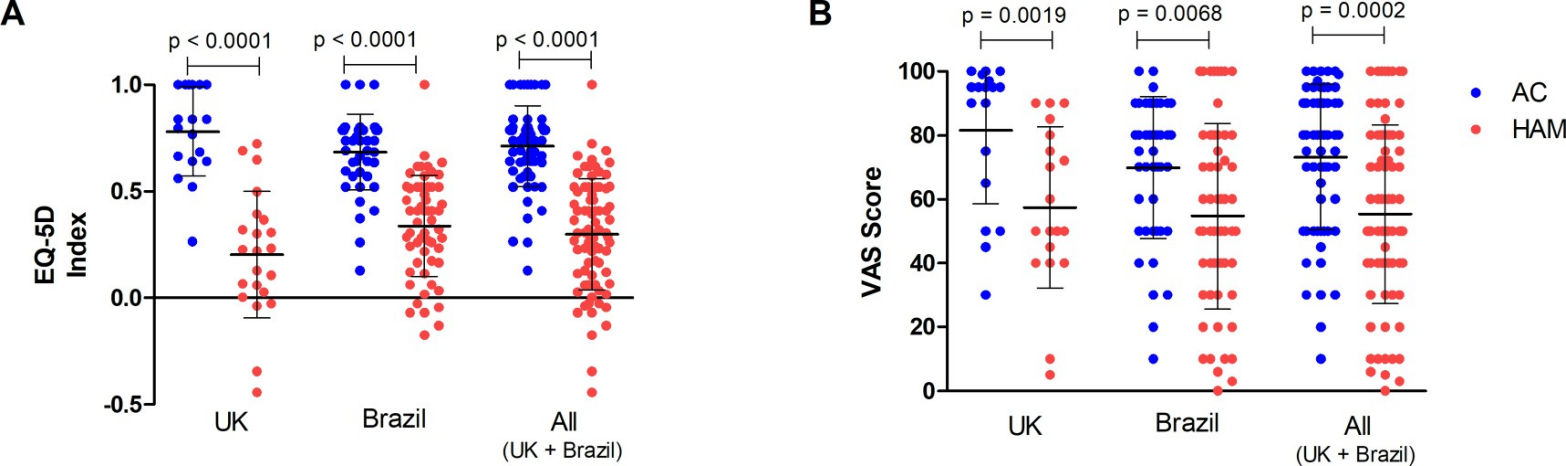
Comparison of EQ-5D Index and VAS Score according to clinical status.

As presented in Fig 2 and Table 3, the average EQ-5D Index score and VAS Score were significantly lower in patients with HAM/TSP (0.3 and 55.9 respectively) than in HTLV-1 asymptomatic carriers (0.7 and 73.2) with 12.7% (n = 10) of patients with HAM/TSP reporting a quality of life considered as worse than death (EQ-5D Index < 0). While patients with HAM/TSP from the UK had an EQ-5D Index score lower than those from Brazil, asymptomatic carriers from the UK had a higher VAS Score than their Brazilian counterparts (Table 3).

**Table 3:** EQ-5D Index and VAS Score for patients with HAM/TSP and in HTLV-1 asymptomatic carriers.

	Brazil	UK	Total
**AC**			
EQ-5D Index	0.6842 (0.177)	0.7802 (0.209)	0.7121 (0.19)
VAS Score	69.9 (22.1)	81.44 (22.9)*	73.2 (22.8)
**HAM/TSP**			
EQ-5D Index	0.3364 (0.236)	0.2025 (0.297)*	0.2991 (0.2597)
VAS Score	54.7 (29)	59.2 (24.2)	55.9 (27.7)

Mean (SD)

* p < 0.05: Brazil vs UK, p < 0.001:HAM vs AC in all groups

AC: Asymptomatic carriers, HAM/TSP: HTLV-1 associated myelopathy / Tropical spastic paraparesis, UK: United Kingdom

Individual Health states, EQ-5D Index and VAS Score from all patients are available as supplementary data.

### Factors impacting QoL in HTLV-1 infected individuals

**Fig 3:**
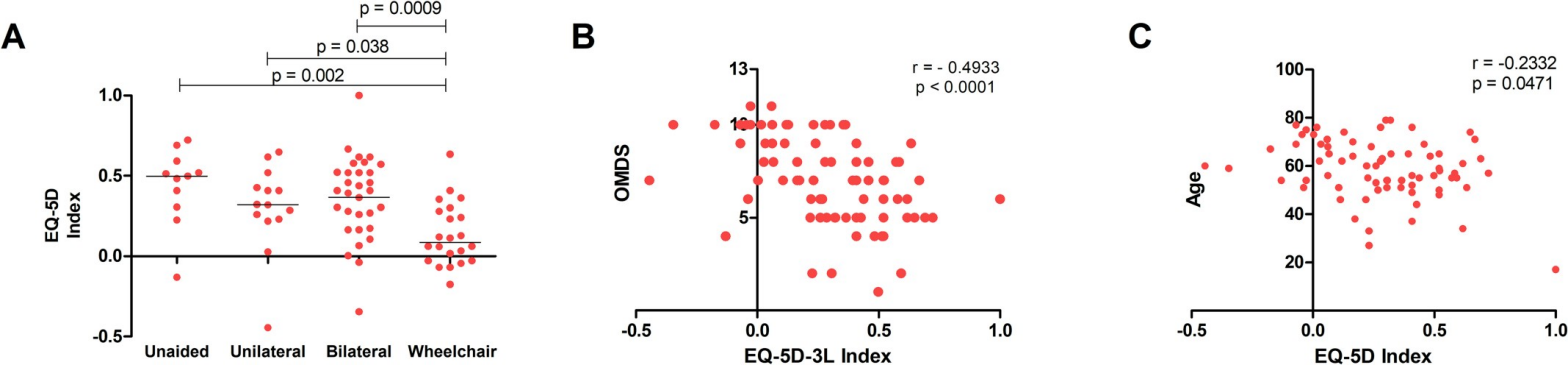
Factors impacting the quality of life in HAM/TSP individuals. A) EQ-5D Index according to the need of support to walk; B) Correlation between OMDS and EQ-5D Index; C) Correlation between EQ-5D Index and age in HAM/TSP individuals.

There was a positive correlation between EQ-5D Index and VAS Score and an inverse correlation between EQ-5D Index and age (Fig 3C). No significant correlation was observed between neither EQ-5D-Index nor VAS Score with duration of symptoms or with educational level overall. No significant difference was observed in EQ-5D Index and VAS Score according to gender in patients with HAM/TSP when considering both Brazil and UK data together, or the UK alone. However, Brazilian female HTLV-1 asymptomatic carriers had a lower EQ-5D Index than Brazilian males (Mean (SD): 0.6224 (0.19) vs 0.7735 (0.1), p = 0.0198). The difference was not significant for VAS Score. QoL in patients restricted to wheelchair was lower than patients who need bilateral or unilateral aid to walk and those who walked unaided (Fig 3A and 3B). EQ-5D Index negatively correlated with OMDS in HAM/TSP patients.

### Impact of immunosuppressive therapy on QoL

In Brazil, the use of intermittent pulsed methyl prednisolone differed according to centre. Where used treatment was administered every 45 days. Patients who had received pulsed methyl prednisolone within the previous 45 days had a higher mean EQ-5D-3L Index (0.4341, SD 0.17) than those that had not (0.242, SD 0.26) p = 0.0015. No difference between VAS Score were observed between these groups (57 (24.2) vs 52.4 (33.3), p = 0.5493). No significant difference between immunosuppressed and not immunosuppressed patients was observed in the UK cohort, however, treatment is offered on a case by case basis with patients with mild or non-progressing disease managed symptomatically.

## Discussion

In an era of many demands on public health and limited resources, cost-effectiveness analyses are increasingly needed. In order to perform an accurate analysis, it is important to have robust data for each input. However, robust health utility values for HAM/TSP have not been reported. Stigum and colleagues conducted a cost-effectiveness analysis of HTLV-1 blood screening in Norway. In their study they attributed a health value for HTLV-1 associated diseases (both HAM/TSP and ATL) of 0.98, the same as for symptomatic HIV, with patients with AIDS having a health value of 0.9 [27]. The values obtained by Stigum were used in further studies [34]. Others used expert opinion to estimate the QALY of HAM/TSP individuals [28] and the values used were 0.06 HAM/TSP, -0.02 acute ATL and 0.81 for indolent ATL. The very wide range in the values used previously indicate that more accurate data is clearly needed. In the present study, the health state utility value was calculated for both patients with HAM/TSP and AC from Brazil and UK using patient data.

**Fig 4:**
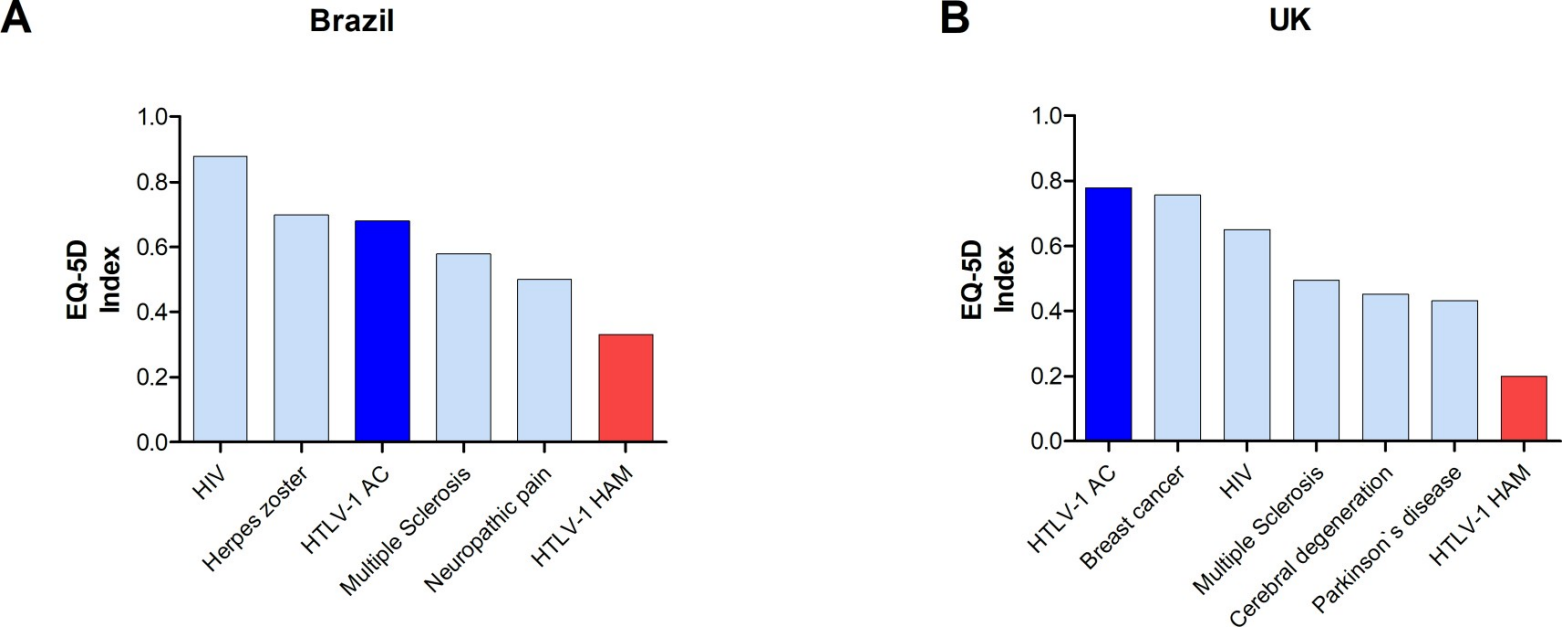
EQ-5D Index in people living with HTLV-1 compared with selected other diseases. A) Brazil, B) UK.

The present study shows that patients with HAM/TSP have an extremely low average EQ-5D Index (<0.3) and about 12% of them reported life to be worse than death (EQ-5D Index < 0). When comparing the UK results with data on more than 130 clinical conditions available in the published UK-based catalogue of EQ-5D index scores for a range of health conditions based on UK preferences, including multiple sclerosis, epilepsy, diabetes mellitus and cerebral degeneration [25], patients with HAM/TSP had the lowest EQ-5D Index (Fig 4). In Brazil, there are only a limited number of studies using the EQ-5D-3L Index and the score for HIV infected patients in the country is reported to be 0.88, with a VAS Score of 80% [35]. This result is much higher than the values reported here for HAM/TSP or even for HTLV-1 AC. Brazilian patients affected with Herpes Zoster (shingles) have a lower EQ-5D-3L Index when compared to baseline (prior to Herpes Zoster rash onset) (0.7 vs 0.9) [36], these values are however higher than those observed here for AC and for patients with HAM/TSP. Interestingly, patients with HAM/TSP also had a lower average for both EQ-5D-3L Index and VAS Score than patients with multiple sclerosis in Brazil (VAS Score Mean (SD) = 67.9, EQ-5D-3L Index Mean (SD) = 0.58 (0.26))[37] or patients with neuropathic pain (EQ-5D-3L Index varying from 0.4–0.6, according to the subtype and VAS score of 47–67.3) [38]. Recently, supporting data were obtained in a Brazilian study where HTLV-1 infected patients had lower QoL than individuals infected with HCV in different domains (physical, vitality, mental health and social functioning in SF-36 questionnaire). They also presented more frequently past major depressive episodes. However, in that study AC and patients with HAM/TSP were not analysed separately [39].

Quality of life is a complex concept that is affected not only by physical health or psychological state. It is also affected by personal beliefs and social relationships. According to the World Health Organization, quality of life comprises the individual’s own perception of their position in life in a broad context that includes culture and value systems in which they are inserted. It is associated to individual`s goals, expectations, standards and concerns [40]. This can explain the differences observed among HTLV-1 infected individuals from different countries.

Previous studies already demonstrated that HTLV-1 infection and HAM/TSP impacts the quality of life of infected individuals but without an adequate score suitable for use in economic analysis and that represents the overall QoL. In HAM/TSP the viral infection is associated with a subsequent inflammatory lesion, mainly at the spinal cord. This may result in multiple clinical presentations that include, but are not limited to, different degrees of weakness, spasticity and sensory disturbance including chronic pain as well as neurogenic bowel and bladder and sexual dysfunction [41]. Added to the physical morbidity, many factors contribute to psychological impairment of affected individuals. They have a sexually transmissible infection which impacts their social relations and family. HAM/TSP is a progressive disease without curative treatment, with a difficult prognosis. Moreover, HTLV-1 infection is a neglected disease increasing their sense of abandonment and their stigma [18,19]. As an incapacitating disease HAM/TSP may also affect patient`s autonomy and their working capacity, impairing their financial condition. Therefore, HAM/TSP impacts patient’s quality of life in a complex way. As expected, the present study confirmed that quality of life is impaired in patients with HAM/TSP but shows that even asymptomatic infection (defined as an absence of any known HTLV-1 associated disease) is associated with a decrease in QoL. Guiltinam et al (1998) already demonstrated that although HTLV-1 infected blood donors were not considered clinically ill they showed signs of subclinical illness, as evaluated by a general well-being questionnaire [42]. Interestingly, the same group showed after approximately 15 years of follow-up that psychological distress (lower general well-being score) at first visit was associated with diagnosis of major depressive disorder and generalised anxiety disorder in the future [43].They also confirmed a higher frequency of major depression and generalised anxiety disorder in HTLV-1/2 seropositive blood donors than seronegative individuals [43]. Recently, a wide range of clinical and neurological symptoms were detected in apparently asymptomatic individuals however it is not known if this constitutes an independent clinical syndrome or whether these are markers of early HAM/TSP [44]. In the present study, most asymptomatic carriers reported some problems in pain/discomfort domain.

Only one previous study was found regarding EQ-5D Index and HTLV-1 infection. Vahidnia and colleagues, analysed the quality of life of 195 recently diagnosed HTLV-1 individuals after blood screening in the United States. EQ-5D index among HTLV infected blood donors were significantly lower than the matched uninfected donors (Mean (SD) = 0.87 (0.16) vs 0.94 (0.10), p < 0.001). In the same study the average VAS score was also lower among HTLV-1 infected individuals (Mean (SD) = 82.6 (16) vs 87.6 (10.6), p < 0.001) [45]. Problems in mobility were reported in 21% of these HTLV-1 infected subjects [45] who, since they were blood donors, were assumed to be healthy [42]. Guiltinan and colleagues (1998) assessed the psychological outcomes in blood donors diagnosed with HTLV-1/2 infection using a general questionnaire (General Well-Being Scale). Seropositive individuals had lower scores than the control group, indicating that they had more psychological distress. HTLV-1 infection remained associated with lower well-being scores even after controlling for many other variables, such as age, race, gender, education, income, donation type, time since notification and intravenous drug use. They hypothesised that the observed results were due to both physical and psychological factors. Asymptomatic patients from Brazil reported more frequently anxiety and depression than UK patients. This could be associated with a better quality of life in the UK, but it can also be associated with differences in how those patients are diagnosed, the support available and their follow-up. While in the UK there is a national referral centre for HTLV-1 supported by the National Health Service, in Brazil there are a limited number of research centres that provide care for some HTLV-1 infected individuals. Moreover, there is a lack of support for recently diagnosed patients in Brazil, differing from the UK.

As reviewed in 2016, pain prevalence among HTLV-1 infected individuals varies from 35–88% and is more commonly reported as persistent [46]. However, pain/discomfort, despite affecting ~85% of patients with HAM/TSP was not the most affected domain, with mobility and usual activities even more frequently reported. It is important to note that all HAM/TSP patients were undergoing symptomatic treatment but pain in HAM/TSP is multifactorial and notoriously difficult to control [47].

Although HAM/TSP is more prevalent in women, no significant difference was observed in quality of life according to gender. A similar picture was described in patients with HAM/TSP using the SF-36 questionnaire [48]. However, among HTLV AC in Brazil, the females had lower EQ-5D-3L Index. In fact, in a recent study conducted in general patients attending the public health system in Brazil, being female was associated with a worse quality of life while having a higher educational level was associated with better quality of life [49]. Educational level did not impact quality of life in the present study.

Regarding the duration of symptoms, patients with more than 10 years of HAM/TSP onset had lower general health when evaluated by SF-36 [48]. As disease progresses, it is possible to observe a deterioration of quality of life. However, patient’s resilience may neutralise this change. Here, no correlation between duration of disease and QoL was observed, however a negative correlation was observed between OMDS and EQ-5D-3L Index, demonstrating that is not the duration of disease but rather its severity that is associated with impaired quality of life. This can also be explained by the different course of disease that can be seen. While some patients can be considered very slow progressors, some individuals may have rapid disease progression [32]. This observation is reinforced by the lower EQ-5D Index values obtained for patients in wheelchair.

In the current study, HIV co-infection was the only exclusion criteria, therefore the possible presence of comorbidities and the fact that HAM/TSP patients were undergoing symptomatic treatment may be considered a limitation of this study. However, as HAM/TSP frequently coexists with other diseases, it can also be considered that the included individuals are truly representative of patients with HAM/TSP. It is also plausible to assume that the quality of life of HAM/TSP individuals without proper follow-up and symptomatic management might be even worse. The observation that patients that were receiving immunosuppressive therapy had a higher QoL than those without immunosuppression is interesting although a placebo effect cannot be excluded and in fact, their own perception of overall health state did not differ between groups, characterised by no significant difference in VAS Score. However, the consensus view is that sustained immunosuppression may be beneficial for HAM/TSP patients [50]. Further studies are needed to evaluate the impact of treatment on the quality of life of patients with HAM/TSP.

In conclusion, this study calculated the health state utility values for patients with HAM/TSP and AC. The impact of HAM/TSP is most severe with patients reporting lower quality of life compared to 130 other diseases [25]. However, the decrease in QoL of the 90% of carriers who are considered asymptomatic individuals is equally important and was observed in both countries. Previous economic analyses have attributed minimal health impact to the asymptomatic carrier state. New health economic analyses are thus urgently required.

## Supporting information

S1 Tables Individual VAS Score, EQ-5D State and EQ-5D Index from all participants. (DOCX)
