# Supplementary material for: Health state utility values in people living with HTLV-1 and in patients with HAM/TSP: The impact of a neglected disease on the quality of life
Source: PLoS Negl Trop Dis. 2020 Oct 16;14(10):e0008761. doi: 10.1371/journal.pntd.0008761 (PMC7592910; doi:10.1371/journal.pntd.0008761)
Supplement: S1 Tables — (DOCX) [file pntd.0008761.s001.docx]

**Supplementary data**

**S1. Tables: Individual VAS Score, EQ-5D State and EQ-5D Index from all participants**

| **HAM/TSP Rio de Janeiro** | | | |
| --- | --- | --- | --- |
| **Patient ID** | **VAS Score** | **EQ-5D State** | **EQ-5D Index** |
| 1 | 30 | 22322 | 0.3213 |
| 2 | 20 | 32333 | -0.0699 |
| 3 | 60 | 32222 | 0.1644 |
| 4 | 20 | 32323 | 0.0332 |
| 5 | 60 | 32211 | 0.2788 |
| 6 | 40 | 32232 | 0.0613 |
| 7 | 100 | 21333 | 0.2852 |
| 8 | 40 | 33333 | -0.1755 |
| 9 | 100 | 32232 | 0.0613 |
| 10 | 30 | 32233 | 0.0164 |
| 11 | 3 | 33332 | -0.1306 |
| 12 | 10 | 32333 | -0.0699 |
| 13 | 50 | 32222 | 0.1644 |
| 14 | 6 | 32223 | 0.1195 |
| 15 | 80 | 21212 | 0.5839 |
| 16 | 40 | 22223 | 0.3627 |
| 17 | 50 | 31223 | 0.2314 |
| 18 | 10 | 22232 | 0.3045 |
| 19 | 100 | 22222 | 0.4076 |
| 20 | 100 | 21123 | 0.5718 |

| **HAM/TSP São Paulo** | | | |
| --- | --- | --- | --- |
| **Patient ID** | **VAS Score** | **EQ-5D State** | **EQ-5D Index** |
| 1 | 75 | 31112 | 0.4379 |
| 2 | 60 | 21122 | 0.6167 |
| 3 | 80 | 21211 | 0.6339 |
| 4 | 80 | 12221 | 0.5777 |
| 5 | 80 | 21122 | 0.6167 |
| 6 | 80 | 22221 | 0.4576 |
| 7 | 50 | 22221 | 0.4576 |
| 8 | 40 | 22222 | 0.4076 |
| 9 | 100 | 21132 | 0.5136 |
| 10 | 50 | 21222 | 0.5195 |
| 11 | 90 | 22233 | 0.2596 |
| 12 | 50 | 22222 | 0.4076 |
| 13 | 50 | 22232 | 0.3045 |
| 14 | 60 | 21121 | 0.6667 |
| 15 | 40 | 21122 | 0.6167 |
| 16 | 20 | 33232 | -0.0443 |
| 17 | 80 | 31321 | 0.24 |

| **HAM/TSP Alagoas** | | | |
| --- | --- | --- | --- |
| **Patient ID** | **VAS Score** | **EQ-5D State** | **EQ-5D Index** |
| 1 | 10 | 22333 | 0.1733 |
| 2 | 30 | 22332 | 0.2182 |
| 3 | 100 | 32211 | 0.2788 |
| 4 | 50 | 22222 | 0.4076 |
| 5 | 70 | 22211 | 0.522 |
| 6 | 40 | 22213 | 0.4271 |
| 7 | 100 | 11111 | 1 |
| 8 | 10 | 22233 | 0.2596 |
| 9 | 0 | 22331 | 0.2682 |
| 10 | 30 | 22222 | 0.4076 |
| 11 | 60 | 21222 | 0.5195 |
| 12 | 40 | 23332 | 0.1126 |
| 13 | 70 | 21222 | 0.5195 |
| 14 | 100 | 33322 | -0.0275 |
| 15 | 70 | 21321 | 0.4832 |
| 16 | 70 | 21222 | 0.5195 |
| 17 | 60 | 22231 | 0.3545 |
| 18 | 50 | 22222 | 0.4076 |
| 19 | 50 | 21222 | 0.5195 |
| 20 | 72 | 12212 | 0.5921 |

| **HAM/TSP UK** | | | |
| --- | --- | --- | --- |
| **Patient ID** | **VAS Score** | **EQ-5D State** | **EQ-5D Index** |
| 1 | 90 | 41244 | 0.231 |
| 2 | 50 | 43432 | 0.393 |
| 3 | 85 | 31221 | 0.723 |
| 4 | 10 | 42544 | 0.003 |
| 5 | 90 | 51311 | 0.3 |
| 6 | 50 | 55512 | -0.028 |
| 7 | 90 | 52322 | 0.059 |
| 8 | 75 | 52222 | 0.066 |
| 9 | 50 | 22221 | 0.648 |
| 10 | 50 | 43452 | -0.038 |
| 11 | 70 | 31331 | 0.691 |
| 12 | 60 | 32441 | 0.32 |
| 13 | 72 | 51232 | 0.128 |
| 14 | 40 | 42342 | 0.306 |
| 15 | 80 | 53455 | -0.445 |
| 16 | 5 | 54553 | -0.346 |
| 17 | 40 | 52233 | 0.027 |
| 18 | 75 | 24432 | 0.365 |
| 19 | 80 | 11242 | 0.498 |
| 20 | 60 | 44441 | 0.226 |
| 21 | 40 | 53413 | 0.106 |
| 22 | 40 | 43442 | 0.221 |

| **AC São Paulo** | | | |
| --- | --- | --- | --- |
| **Patient ID** | **VAS Score** | **EQ-5D State** | **EQ-5D Index** |
| 1 | 100 | 11111 | 1 |
| 2 | 50 | 31233 | 0.1283 |
| 3 | 30 | 22233 | 0.2596 |
| 4 | 10 | 11121 | 0.7868 |
| 5 | 30 | 21233 | 0.3715 |
| 6 | 80 | 11121 | 0.7868 |
| 7 | 90 | 11112 | 0.8012 |
| 8 | 80 | 21211 | 0.6339 |

| **AC Alagoas** | | | |
| --- | --- | --- | --- |
| **Patient ID** | **VAS Score** | **EQ-5D State** | **EQ-5D Index** |
| 1 | 90 | 11121 | 0.7868 |
| 2 | 90 | 11112 | 0.8012 |
| 3 | 90 | 11113 | 0.7563 |
| 4 | 80 | 11222 | 0.6396 |
| 5 | 75 | 11223 | 0.5947 |
| 6 | 70 | 21222 | 0.5195 |
| 7 | 80 | 11121 | 0.7868 |
| 8 | 50 | 21121 | 0.6667 |
| 9 | 70 | 21222 | 0.5195 |
| 10 | 90 | 11221 | 0.6896 |
| 11 | 50 | 22222 | 0.4076 |
| 12 | 80 | 11121 | 0.7868 |
| 13 | 50 | 21221 | 0.5695 |
| 14 | 75 | 11111 | 1 |
| 15 | 40 | 11122 | 0.7368 |
| 16 | 90 | 11112 | 0.8012 |
| 17 | 95 | 11121 | 0.7868 |
| 18 | 80 | 11111 | 1 |
| 19 | 20 | 21113 | 0.6362 |
| 20 | 50 | 11222 | 0.6396 |
| 21 | 70 | 11133 | 0.5888 |
| 22 | 40 | 11122 | 0.7368 |
| 23 | 70 | 11121 | 0.7868 |
| 24 | 60 | 11332 | 0.4502 |
| 25 | 60 | 21222 | 0.5195 |
| 26 | 80 | 11122 | 0.7368 |
| 27 | 50 | 11131 | 0.6837 |
| 28 | 80 | 11221 | 0.6896 |
| 29 | 80 | 11121 | 0.7868 |
| 30 | 70 | 11122 | 0.7368 |
| 31 | 70 | 21221 | 0.5695 |
| 32 | 100 | 11112 | 0.8012 |
| 33 | 90 | 11122 | 0.7368 |
| 34 | 90 | 11112 | 0.8012 |
| 35 | 90 | 11121 | 0.7868 |
| 36 | 90 | 11112 | 0.8012 |

| **AC UK** | | | |
| --- | --- | --- | --- |
| **Patient ID** | **VAS Score** | **EQ-5D State** | **EQ-5D Index** |
| 1 | 100 | 11111 | 1 |
| 2 | 100 | 11151 | 0.264 |
| 3 | 30 | 21223 | 0.664 |
| 4 | 95 | 11121 | 0.837 |
| 5 | 75 | 21233 | 0.639 |
| 6 | 95 | 11111 | 1 |
| 7 | 90 | 21121 | 0.767 |
| 8 | 90 | 42323 | 0.521 |
| 9 | 95 | 11121 | 0.837 |
| 10 | 50 | 22332 | 0.56 |
| 11 | 50 | 21132 | 0.683 |
| 12 | 45 | 21233 | 0.639 |
| 13 | 97 | 11111 | 1 |
| 14 | 99 | 11111 | 1 |
| 15 | 100 | 11111 | 1 |
| 16 | 65 | 11111 | 1 |
| 17 | 95 | 11121 | 0.837 |
| 18 | 95 | 11221 | 0.795 |
